# Is transperitoneal laparoscopic adrenalectomy for pheochromocytoma really more challenging? A propensity score-matched analysis

**DOI:** 10.1007/s40618-023-02013-7

**Published:** 2023-01-27

**Authors:** D. Corallino, A. Balla, L. Palmieri, I. Sperduti, M. Ortenzi, M. Guerrieri, A. M. Paganini

**Affiliations:** 1 Department of General Surgery and Surgical Specialties, Sapienza University of Rome, Viale del Policlinico 155, 00161 Rome, Italy; 2 UOC of General and Minimally Invasive Surgery, Hospital “San Paolo”, Largo Donatori del Sangue 1, 00053 Civitavecchia, Rome, Italy; 3 Department of Biostatistics, IRCCS Regina Elena National Cancer Institute, Via Elio Chianesi 53, 00144 Rome, Italy; 4 Department of General Surgery, Università Politecnica Delle Marche, Piazza Roma 22, 60121 Ancona, Italy

**Keywords:** Pheochromocytoma (PHEO), Adrenal lesions, Transperitoneal laparoscopic adrenalectomy (TLA), Open adrenalectomy (OA), Propensity score matching (PSM)

## Abstract

**Purpose:**

Minimally invasive surgery is the gold standard treatment for adrenal masses, but it may be a challenging procedure in the case of pheochromocytoma (PHEO). The aim of the present study is to report the results of transperitoneal laparoscopic adrenalectomy (TLA) in cases of PHEO in comparison to other types of adrenal lesions.

**Methods:**

From 1994 to 2021, 629 patients underwent adrenalectomy. Twenty-two and thirty-five patients, respectively, were excluded because they underwent bilateral and open adrenalectomy, leaving 572 patients for inclusion. Of these, 114 patients had PHEO (Group A), and 458 had other types of lesions (Group B). To adjust for potential baseline confounders, a propensity score matching (PSM) analysis was conducted.

**Results:**

After PSM, 114 matched pairs of patients were identified from each group. Statistically significant differences were not observed when comparing the median operative time (85 and 90 min in Groups A and B, respectively, *p* = 0.627), conversion rate [6 (5.3%) in each group, *p* = 1.000], transfusion rate [4 (3.5%) and 3 (2.6%) in Groups A and B, respectively, *p* = 1.000], complication rate [7 (6.1%) and 9 (7.9%) in Groups A and B, respectively, *p* = 0.796), median postoperative hospital stay (3.9 and 3.6 days in Groups A and B, respectively, *p* = 0.110), and mortality rate [1 (0.9%) in each group, *p* = 1.000].

**Conclusions:**

Based on this analysis, the results of TLA for PHEO are equivalent to those of TLA for other types of adrenal lesions, but the fundamental requirements are multidisciplinary patient management and adequate surgeon experience. Further prospective studies are required to draw definitive conclusions.

## Introduction

Pheochromocytoma (PHEO) is a rare catecholamine-producing neuroendocrine tumour arising from chromaffin cells of the adrenal medulla [1–3]. PHEO is a life-threatening condition due to excessive catecholamine production and is characterized by clinical symptoms, including sweating, headaches, palpitations, perspiration, tremors, and facial pallor [4, 5]. These symptoms are often paroxysmal or can be induced by a variety of events, such as strenuous physical exertion, delivery, trauma, anaesthesia, and surgery [4, 5].

In patients with PHEO, adrenalectomy is the gold standard treatment, although resection may be challenging for surgeons due to a high risk of intraoperative haemodynamic instability from excessive catecholamine release during the induction of anaesthesia and with intraoperative surgical manipulation of the gland [6–9].

Since the first report of transperitoneal laparoscopic adrenalectomy (TLA) in 1992 [10], minimally invasive surgery (MIS) has largely replaced open adrenalectomy (OA) as the standard procedure, but its role in the case of large PHEOs and cancer is still debated [6, 11–14].

Although laparoscopic adrenalectomy (LA) and robotic adrenalectomy (RA) versus OA for the management of PHEO have been reported to be associated with better postoperative outcomes in terms of lower estimated blood loss, a shorter hospital stay, and less haemodynamic instability [15, 16], the effectiveness of MIS compared to OA for PHEO is still controversial [11, 17]. Furthermore, the influence of tumour type and activity on postoperative outcomes has not yet been well established [18].

Several studies have compared the postoperative outcomes after LA for PHEO versus other types of adrenal lesions [18, 19]. Some authors reported that PHEO and cortisol-producing adenoma are associated with more demanding surgery and poor postoperative outcomes [19–22]. Other authors reported no differences in postoperative complications based on the type of adrenal lesions [18, 23–25]. However, most of these comparative studies include a relatively small number of patients and a paucity of postoperative complications [18–21, 23–26]. The aim of the present propensity score-matched (PSM) analysis is to compare the 30-day surgical outcomes of TLA for PHEO compared to other types of adrenal lesions.

## Materials and methods

This study is a retrospective analysis of prospectively collected data. Institutional review board approval (number: 1/2022) and informed consent were obtained from all participants.

From 1994 to 2021, 629 patients underwent adrenalectomy in two centres (Department of General Surgery and Surgical Specialties, Policlinico Umberto I, Sapienza University of Rome, and Department of General Surgery, Università Politecnica delle Marche, Ancona, Italy).

**Fig. 1:**
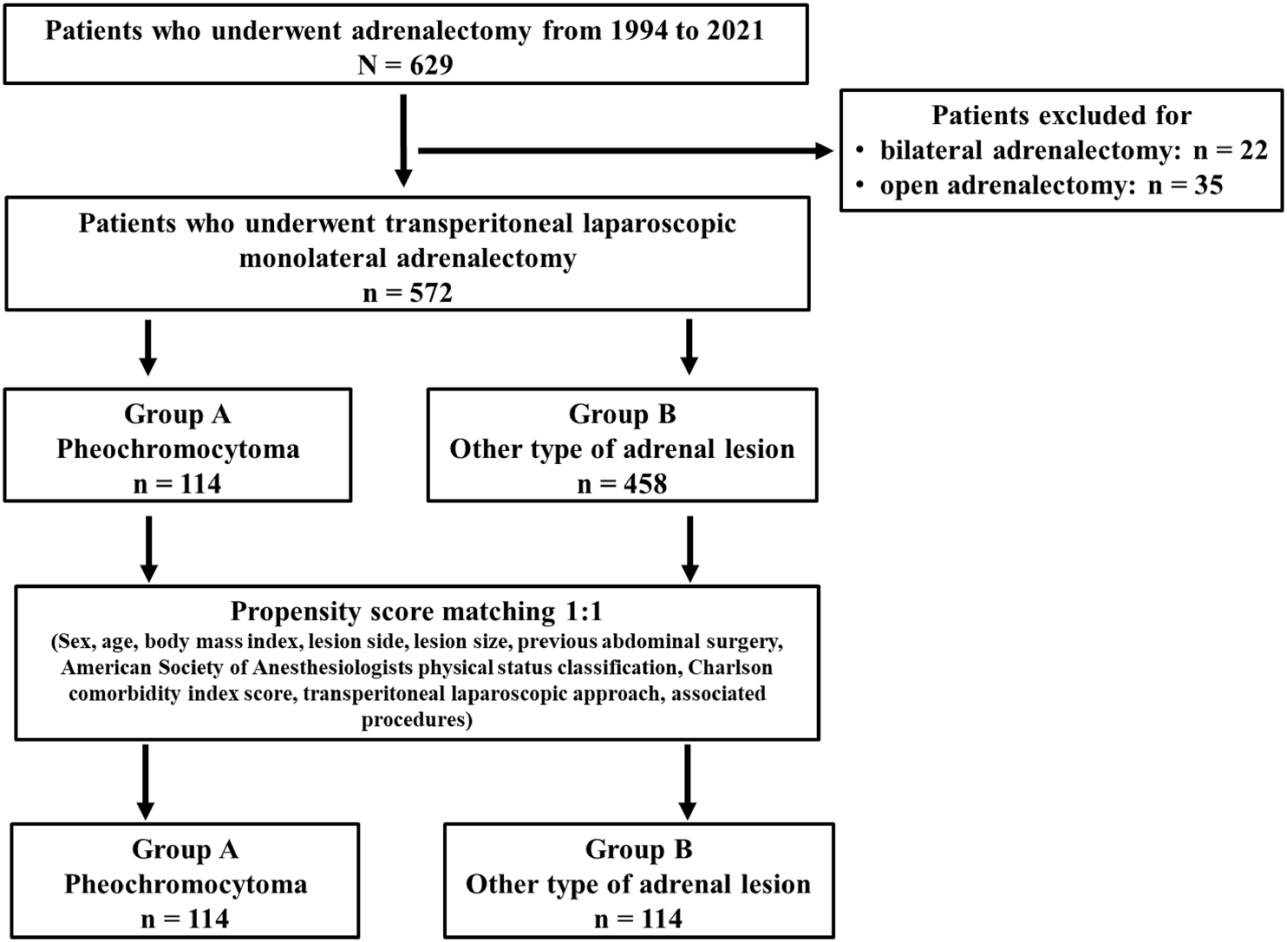
Patient selection

Patients who underwent bilateral [29] and OA were excluded (22 and 35 patients, respectively), leaving 572 patients for inclusion in the study (Fig. 1).

Out of the 572 patients, 114 (19.9%) and 458 (80.1%) patients underwent LA for PHEO and for other types of lesions, respectively (Fig. 1).

In all patients, the same medical treatment protocol was followed (which has changed over the years in accordance with the guidelines), and they underwent an identical surgical approach (which, on the contrary, has remained unchanged over the years), as previously reported [27–32].

The patients were evaluated together with the endocrinologist and anaesthesiologist consultants by physical examination and hormonal assessment, which currently includes plasma free or urinary fractionated metanephrines to exclude PHEO, 1 mg overnight dexamethasone suppression test to exclude cortisol excess, aldosterone/renin ratio to exclude primary aldosteronism in case of concomitant hypertension or unexplained hypokalaemia and measurement of sex hormones and steroid precursors in case of clinical or imaging features suggestive of adrenocortical carcinoma, as recommended [1, 17]. Magnetic resonance imaging (MRI) and computerized tomography (CT) scans were performed for each patient [30–32]. Scintigraphy with ^131^I-metabenzilguanidine or 6-^18^F-fluoro-l-dopa positron emission tomography was performed in any case of equivocal CT and MRI or discrepancy between imaging and biochemical tests [30–32].

A total body CT scan was performed for all patients who had PHEO.

Last, in all the patients with secreting adrenal lesions, arterial hypertension, chronic heart disease and electrocardiographic abnormalities, echocardiography was performed to rule out any underlying cardiac disease, according to our endocrinological and anaesthesiological protocols.

In the case of PHEO, the goals of perioperative management have always been blood pressure control and normalization of intravascular volume, although in this study, this has been achieved with different protocols over the years [30–32].

Currently, all patients with PHEO start preoperative pharmacological preparation with alpha-blockers (doxazosin: starting dose 2 mg/day) 14 days before surgery. If the patient is not affected by hypertension, the patient still starts with a low dose of an alpha-blocker, which is then modulated by the endocrinologists based on the blood pressure levels, and the final dose can be as high as 32 mg/day, according to the current guidelines [17, 30]. Beta-blockers (atenolol: starting dose 25 mg/day, final dose 50 mg/day) are added in case of tachycardia episodes, after at least three days of alpha blockade administration, to avoid hypertensive crises due to the beta blockade without alpha blockade [17, 30].

The preoperative treatment also includes administration of normal saline 2000 cc the evening before surgery (500 or 1000 cc in patients with heart or renal failure) to reverse catecholamine-induced blood volume contraction and to prevent severe hypotension after tumour removal, as previously reported and as recommended [17, 30–32].

Blood pressure, heart rate, and blood glucose level monitoring with adjustment of associated therapies were performed in the immediate postoperative period, as recommended [17, 30–32], and these were administered based on close collaboration with the anaesthesiology team.

### Study design

Patient characteristics [including sex, age and body mass index (BMI)], American Society of Anaesthesiologists (ASA) grade, Charlson comorbidity index (CCI) score [33], previous abdominal surgery, lesion side, type of TLA (anterior or flank approach on the right, anterior, flank or submesocolic retropancreatic approach on the left), associated procedures, conversion rate, operative time, intra- and 30-day postoperative complications (graded according to the Clavien–Dindo classification [34]), adrenal lesion size and histology, hospital stay and 30-day mortality were stored in a Microsoft Excel program (Microsoft Corporation, Redmond, Washington, USA).

### Statistical analysis

Continuous data are expressed as medians and 95% confidence intervals (CIs), while categorical variables are expressed as frequencies and percentages. The Mann–Whitney *U* test and Fisher’s exact test were used for the comparisons between the groups for continuous and categorical variables, respectively. A *p* value lower than 0.05 was considered statistically significant. Statistical analyses were carried out with SPSS software 22.0 (SPSS Inc., Chicago, Illinois, USA).

To minimize the selection bias and potential confounding factors in this retrospective cohort study, a propensity score matching (PSM) analysis 1:1 was performed to match the Group A patients with the Group B patients. A logistic regression model was applied to evaluate the propensity score of each patient according to the following covariables: sex, age, BMI, ASA grade, CCI score, lesion side, lesion size, previous abdominal surgery, type of TLA, and associated procedures. Two well-matched patient cohorts were obtained using a 1:1 nearest neighbour matching algorithm that pairs patients with the closest PS.

## Results

The patient characteristics and surgical outcomes of the entire series after PSM comparing Group A versus Group B are reported in Table 1.

**Table 1:** Results after propensity score matching

	Group A	Group B	*p* value
	*n* = 114 (50%)	*n* = 114 (50%)	
Sex ratio, *n* (%)			
- Men	49 (43)	35 (30.7)	0.074
- Women	65 (57)	79 (69.3)	
Median age, years (CI 95%)	52.5 (49.3–55.1)	53 (50.7–56.4)	0.706
Median body mass index, kg/m^2^ (CI 95%)	25.5 (24.7–26.4)	26 (26.2–28.2)	0.714
ASA grade, *n* (%)			
- I–II	93 (81.6)	90 (78.9)	0.618
- III–IV	21 (18.4)	24 (21.1)	
CCI score, *n* (%)			
- 0–1	61 (53.5)	60 (52.6)	0.894
- ≥ 2	53 (46.5)	54 (47.4)	
Previous abdominal surgery, *n* (%)	8 (7)	6 (5.3)	0.784
Lesion side, *n* (%)			
- Right	63 (55.3)	70 (61.4)	0.420
- Left	51 (44.7)	44 (38.6)	
TLA, *n* (%)			
- Anterior	69 (60.5)	80 (70.2)	0.164
- Left anterior submesocolic retropancreatic	45 (39.5)	33 (28.9)	0.124
- Flank	–	1 (0.9)	0.122
Associated procedures, *n* (%)	4 (3.5)	5 (4.4)	1.000
Conversion rate, *n* (%)	6 (5.3)	6 (5.3)	1.000
- Adhesions from previous surgery	1 (0.9)	1 (0.9)	1.000
- Adhesion to pancreas	–	–	1.000
- Adhesion to liver	1 (0.9)	1 (0.9)	1.000
- Bleeding	2 (1.8)	2 (1.8)	1.000
- Respiratory failure from pneumoperitoneum	1 (0.9)	1 (0.9)	1.000
- Retrocaval mass growth	1 (0.9)	1 (0.9)	1.000
Median operative time, minutes (CI 95%)	85 (88.6–105.9)	90 (92–112.5)	0.627
Postoperative complications, *n* (%, Clavien–Dindo classification grade)	10 (8.8)	10 (8.8)	1.000
Surgical complications	7 (6.1)	6 (5.3)	1.000
- Acute urinary retention	–	–	1.000
- Ileus	–	–	1.000
- Abdominal abscess	–	–	1.000
- Anaemia	4 (3.5, II)	3 (2.6, II)	1.000
- Fever	1 (0.9, II)	2 (1.8, II)	1.000
- Wound infection	1 (0.9, II)	1 (0.9, II)	1.000
- Colonic fistula	–	–	1.000
- Chylous ascites	1 (0.9, III-a)	–	1.000
- Hemoperitoneum	–	–	1.000
Medical complications	3 (2.6)	4 (3.5)	1.000
- Pleural effusion	1 (0.9, I)	1 (0.9, I)	1.000
- Pneumonia	2 (1.8, II)	2 (1.8, I)	1.000
- Atrial fibrillation	–	–	1.000
- Acute myocardial infarction	–	1 (0.9, II)	1.000
Median lesion size at definitive histology, cm (CI 95%)	4.3 (4–4.7)	4.1 (3.9–4.8)	0.151
Definitive histology (benign: malignant), *n* %			
Benign lesions	101 (88.6)	100 (87.7)	1.000
- Pheochromocytoma	101 (88.6)	–	** < 0.001**
- Secreting adenoma	–	40 (35.1)	** < 0.001**
- Non-secreting adenoma	–	30 (26.3)	** < 0.001**
- Hyperplasia	–	18 (15.8)	** < 0.001**
- Myelolipoma	–	10 (8.8)	**0.002**
- Adrenal cyst	–	2 (1.8)	0.450
- Angiomyolipoma	–	–	1.000
Malignant lesions	13 (11.4)	14 (12.3)	1.000
- Pheochromocytoma	13 (11.4)	–	** < 0.001**
- Adrenal carcinoma	–	10 (8.8)	0.002
- Adrenal metastases	–	4 (3.5)	0.123
Median hospital stay, days (CI 95%)	4 (4.1–5)	4.1 (3.6–4.5)	0.110
Mortality, *n* (%)	1 (0.9)	1 (0.9)	1.000

*ASA* American Society of Anaesthesiologists, *CCI* Charlson comorbidity index, *TLA* transperitoneal laparoscopic adrenalectomy, *CI* confidence intervals

Statistically significant differences in bold

After PSM, two homogeneous groups were obtained, without any statistically significant differences regarding the preoperative characteristics.

Comparing the postoperative outcomes, no statistically significant differences were observed in median operative time (85 and 90 min in Groups A and B, respectively, *p* = 0.627), conversion rate [6 (5.3%) in each group, *p* = 1.000], transfusion rate [4 (3.5%) and 3 (2.6%) in Groups A and B, respectively, *p* = 1.000], complication rate [7 (6.1%) and 9 (7.9%) in Groups A and B, respectively, *p* = 0.796], median postoperative hospital stay (3.9 and 3.6 days in Groups A and B, respectively, *p* = 0.110), and mortality rate [1 (0.9%) in each group, *p* = 1.000].

Concerning the type of TLA, the anterior approach was mostly used in both groups [69 cases (61%) versus 80 cases (70%), *p* = 0.164], followed by the left submesocolic retropancreatic approach [45 cases (40%) versus 33 cases (29%), *p* = 0.0124], while the flank approach was the least adopted in both groups [0 cases versus 1 case (1%), *p* = 0.122].

Comparing the incidence of metastatic versus benign lesions at the time of surgery, statistically significant differences were not observed between the two groups. A negative resection margin was achieved in all cases.

An associated surgical procedure was performed in four patients (3.5%) in Group A [cholecystectomy in 3 (2.6%) and pedunculated uterine fibroid resection in 1 (0.9%) patients] and in three patients (2.6%) in Group B [cholecystectomy in 1 (0.9%) and umbilical hernia repair in 2 (1.8%) patients].

In Group A, one (0.9%) human immunodeficiency virus (HIV)-positive, ASA III, male patient, who had undergone a previous coronary artery bypass graft died from an acute myocardial infarction on postoperative Day 4. In Group B, 1 (0.9%) female patient with ASA III died from acute respiratory failure on postoperative Day 4.

## Discussion

The present PSM analysis demonstrates that TLA for PHEO is associated with the same 30-day surgical outcomes as for other types of lesions, with no statistically significant differences.

As reported in the literature some preoperative characteristics (high BMI, previous abdominal surgery, and large adrenal masses) are related to a higher risk of intra- and postoperative complications [12, 14, 35]. Therefore, a PSM analysis was conducted to balance potential confounders and to obtain two homogeneous groups.

As reported in the European Society for Medical Oncology (ESMO) guidelines, MIS is the gold standard treatment in cases of PHEO up to 6 cm. However, OA is still recommended if complete resection cannot be achieved by MIS [1, 17]. Some authors report that LA for PHEO might result in higher intra- and postoperative complication rates, more blood loss, a longer operative time and a longer hospital stay, but these issues are still debated [19, 26, 36, 37]. The present analysis, however, does not support these words of caution, and our results are in line with those reported by other authors [7, 11, 24, 38]. In a recent study by Arolfo et al*.*, including 114 patients, MIS has showed to be safe and effective even in cases of PHEOs greater than 5 cm in diameter [38]. A recent meta-analysis by Li et al., including more than 700 patients, also reported the superiority of LA compared to OA in the case of PHEO in terms of estimated blood loss, transfusion rate, haemodynamic instability, postoperative complication rate, and length of hospital stay [11]. Regardless, this meta-analysis included only one randomized controlled trial, and it compared heterogeneous patient groups, as the tumour size was smaller [11]. However, the BMI was higher in the LA group than in the OA group [11]. Moreover, some preoperative variables, such as previous abdominal surgery and associated procedures, are missing, which might influence the surgical outcomes [11].

It should be emphasized that in this study the most commonly used approach of TLA was the anterior approach. As reported in the literature [20, 25, 26], early identification and ligation of the adrenal vein performed prior to any gland manipulation and careful dissection with no adrenal capsule disruption are the recommended strategies to reduce the intraoperative catecholamine release, and this represents one of the main advantages of the anterior and submesocolic TLA [22, 27, 30–32].

Since there is no clear superiority of one approach over another [39–42], the recommendations of the Society of American Gastrointestinal and Endoscopic Surgeons (SAGES) suggest that the surgeon should employ the surgical approach for LA that he or she is most familiar with [43]. For this reason, even if TLA by the anterior approach is an uncommon procedure worldwide, in our experience we have continued to use it. For us, it is more familiar in comparison to other approaches, and our results are similar to those reported in the literature by other authors using the lateral or posterior approaches in terms of operative time, conversion rates and morbidity rates [22, 27, 30–32, 39–41].

These data suggest that, regardless of the adopted approach, the fundamental requirements are adequate surgical experience in advanced MIS and multidisciplinary patient management in high-volume centres [1, 44–50].

Our study suggests that TLA for PHEO using the transperitoneal anterior and submesocolic approach is as safe and effective as TLA performed for other types of tumours. This further supports the concept that other preoperative parameters are probably useful to predict the difficulty of laparoscopic adrenalectomy, such as sex, previous surgery, BMI, site and size of the lesion, associated procedures, and comorbidities [12, 35, 37, 51–53].

The main limitations of the present study are its retrospective nature and the lack of data regarding intraoperative monitoring values for blood pressure, heart rate and glucose in all patients. The long study period also represents a limitation as it involves a certain heterogeneity of the study population, since the recommendations in the management of patients with adrenal lesions have changed considerably over the years [17, 53–56]. The diagnosis and management of patients with adrenal lesions differed between the patients treated in the first and last years of the present study (e.g. the diagnosis of PHEO was initially based on catecholamines and vanillylmandelic acid, while currently, it is based on urinary or plasma metanephrines [17, 55, 56]. Also, our perioperative medical management has undergone changes over the years [13, 22, 30–32]). However, this heterogeneity concerns both the study and the control group; moreover, we did not find differences with regard to the surgical approach over the years which is the real purpose of the study. Furthermore, to the best of our knowledge, the present study is the first and largest one comparing the surgical outcomes of LA performed for PHEO versus other types of lesions, and this study used PSM analysis to account for potential confounders.

In conclusion, based on the present study, the surgical outcomes of TLA for PHEO are equivalent to those of TLA for other types of tumours with the same preoperative characteristics. Adequate surgical experience in advanced MIS and disease-specific LA, coupled with a multidisciplinary patient management including consultations with the endocrinologist and the anaesthesiologist, are essential requirements. Further prospective randomized controlled trials with a larger number of patients are required to draw definitive conclusions.

## Data Availability

The datasets generated analysed during the current study are available from the corresponding author on reasonable request.
